# A Rapid Recombinase Polymerase Amplification–CRISPR/Cas12a Assay for Detecting Grapevine Black-Foot Pathogens

**DOI:** 10.3390/jof12070455

**Published:** 2026-06-23

**Authors:** Wenwen Liang, Baoyu Wang, Junbo Peng, Caiping Huang, Yueyan Zhou, Xing Li, Wei Zhang, Jiye Yan

**Affiliations:** 1 College of Plant Protection, Hebei Agricultural University, 2596 Lekai South Street, Lianchi District, Baoding 071000, China; lww9868@163.com (W.L.); jiyeyan@vip.163.com (J.Y.); 2 Beijing Key Laboratory of Environment Friendly Management on Fruit Diseases and Pests in North China, Institute of Plant Protection, Beijing Academy of Agriculture and Forestry Sciences, Beijing 100097, China; baoyu.wang@etu.univ-cotedazur.fr (B.W.); pjb169961@163.com (J.P.); huangcaiping@baafs.net.cn (C.H.); yueyan_zhou@163.com (Y.Z.)

**Keywords:** grapevine black-foot disease, *Ilyonectria*, *Dactylonectria*, RPA, CRISPR/Cas12a, lateral flow assay, rapid diagnosis

## Abstract

Grapevine black-foot disease is a destructive trunk disease with a complex pathogen composition that often involves mixed and latent infections, making timely field diagnosis challenging. To improve rapid field detection, we developed a rapid, sensitive, and low instrument-dependent nucleic acid assay. The assay integrates recombinase polymerase amplification (RPA) and clustered regularly interspaced short palindromic repeats (CRISPR)–Cas12a for the detection of *Ilyonectria* and *Dactylonectria*, two genera associated with grapevine black-foot disease. Conserved regions of the histone H3 and β-tubulin genes were selected for the design of specific RPA primers and corresponding CRISPR RNAs (crRNAs) for *Ilyonectria* and *Dactylonectria*, respectively. A workflow integrating RPA, Cas12a-mediated recognition, and lateral flow assay (LFA)-based visualization was established. The reaction conditions were optimized to enhance amplification efficiency and Cas12a recognition stability. Specificity was evaluated using DNA from target and non-target fungi, and sensitivity was determined using serially diluted templates. Under optimized conditions, the assay detected *Ilyonectria* DNA at concentrations as low as 3.6 ng/μL within 1 h at 39 °C. For *Dactylonectria*, the detection limit reached 80 fg/μL within 50 min at 41 °C. No cross-reactivity was observed. The LFA strips exhibited positive and negative bands within minutes, enabling rapid visual interpretation. This RPA-CRISPR/Cas12a-LFA system provides a rapid, visually interpretable approach for detecting selected grapevine black-foot disease-associated species in China. The workflow reduces the requirement for specialized thermocycling and fluorescence detection equipment during amplification and readout, following DNA extraction.

## 1. Introduction

Grapevine trunk diseases are among the most destructive diseases affecting grape production worldwide, causing vine decline, reduced yield and quality, and significant economic losses [1,2]. These diseases are caused by diverse fungal pathogens and frequently involve mixed and latent infections, complicating rapid detection. Field symptoms often overlap among co-occurring trunk diseases and resemble those caused by abiotic stressors, making accurate identification difficult in the early stages [3,4,5,6].

Among grapevine trunk diseases, black-foot disease is particularly destructive in nurseries and newly established vineyards [7,8]. Surveys have reported high incidence in commercial nurseries. For example, approximately 39% of nursery plants were infected in Uruguay, and pathogens associated with black-foot disease were detected in 38.1% of surveyed nurseries in Turkey [9,10]. Infection causes the rapid decline or death of young vines and shortens vineyard lifespan [11]. The pathogens causing black-foot disease are soilborne and can be spread via irrigation and agricultural practices.

In China, at least 10 species belonging to 5 genera have been reported as the causal agents of black-foot disease: *Dactylonectria*, *Ilyonectria*, *Cylindrocarpon*, *Campylocarpon*, and *Neonectria* [2,12,13,14,15]. Among the black-foot disease-associated genera identified in a recent survey covering multiple grape-growing regions in China, *Dactylonectria* and *Ilyonectria* showed the broadest geographic distributions [16]. Given the pathogen diversity and difficulty of species-level discrimination under field conditions, detection of selected species within these genera associated with black-foot disease in China provides a practical diagnostic strategy.

Traditional diagnosis relies on pathogen isolation, morphological characterization, and molecular identification, which are time-consuming and require laboratory facilities [17,18]. PCR-based methods, including conventional PCR, quantitative real-time PCR (qPCR), droplet digital PCR, nested PCR, and multiplex PCR, have been developed to detect black-foot pathogens [19,20,21,22,23]. However, these methods require thermal cyclers or fluorescence detection platforms, which limits their application in field and resource-limited settings.

Recombinase polymerase amplification (RPA) enables rapid DNA amplification under isothermal conditions (37–41 °C) in 10–40 min and does not require complex instrumentation [24]. Clustered regularly interspaced short palindromic repeats (CRISPR)-Cas12a provides highly specific target recognition and exhibits collateral trans-cleavage activity upon target binding. This activity enables signal amplification using labeled single-stranded DNA reporters [25]. The combination of RPA and CRISPR/Cas12a coupled with a lateral flow assay (LFA) is a promising approach for rapid, sensitive, and instrument-independent pathogen detection [26,27,28,29].

Despite advances in molecular diagnostics, a rapid, sensitive, and visually interpretable detection system for grapevine black-foot disease remains to be established. Therefore, this study aimed to develop an RPA–CRISPR/Cas12a-based rapid detection system with two primer/crRNA sets to separately detect selected species within *Ilyonectria* and *Dactylonectria* associated with grapevine black-foot disease in China. The defined target range comprises *Ilyonectria liriodendri*, *Dactylonectria alcacerensis*, *D. macrodidyma*, *D. novozelandica*, and *D. torresensis*. LFA was used for visual readout. By designing specific RPA primers and crRNAs and systematically optimizing the amplification and Cas12a reaction conditions, we established a rapid and visually interpretable detection method requiring less specialized instrumentation than PCR-based methods. In addition, field-collected symptomatic grapevine samples were used to evaluate the applicability of the established *Ilyonectria*- and *Dactylonectria*-targeted RPA–CRISPR/Cas12a–LFA detection systems.

## 2. Materials and Methods

### 2.1. Fungal Strains and Genomic DNA Extraction

A total of 30 fungal strains obtained from the Institute of Plant Protection, Beijing Academy of Agriculture and Forestry Sciences (Beijing, China), were used in this study (Table 1). The strains were cultured on potato dextrose agar at 25 °C for 7–14 days in an artificial climate chamber. Mycelia were harvested, and genomic DNA was extracted using the conventional cetyltrimethylammonium bromide method. The DNA quality and concentration were assessed using agarose gel electrophoresis and a NanoDrop 2000 spectrophotometer (Thermo Fisher Scientific, Waltham, MA, USA). Genomic DNA was diluted to the required concentrations with double-distilled water (ddH_2_O) and stored at −20 °C until use.

### 2.2. RPA Primer and crRNA Design and Reporter Molecule Synthesis

The *histone H3* (*HIS*) and *β-tubulin* (*TUB*) gene sequences of grapevine black-foot pathogens and other grape-associated fungal pathogens were retrieved from the National Center for Biotechnology Information database (https://www.ncbi.nlm.nih.gov/, accessed on 15 August 2024.). Multiple sequence alignment was performed using the MAFFT online service (v7) [34]. Based on the alignment results, nucleotide variations between target and non-target species were identified, and discriminatory regions were selected as candidate amplification targets.

Primer design followed the TwistDx RPA primer design guidelines (TwistAmp^®^ DNA Amplification Kits Assay Design Manual, Part number INASDM). Target regions with a GC content of 40–60% were selected, primers were 30–35 bp in length, and the optimal amplicon length was 100–200 bp (maximum < 500 bp). Candidate primers were evaluated with Oligo 7 software (v7.60; Molecular Biology Insights, Inc., Cascade, CO, USA) to eliminate sequences prone to secondary structures or primer-dimer formation. Primer specificity was analyzed using Primer-BLAST (2.16.0) (https://www.ncbi.nlm.nih.gov/tools/primer-blast/, accessed on 4 June 2026) against sequences available in the NCBI database to assess the predicted amplification range of each primer pair.

After the selection of optimal RPA primers, crRNAs were designed in the corresponding amplicon regions. As canonical protospacer adjacent motif (PAM) sequences (5′-TTTV-3′) were absent in the selected regions, suboptimal PAM sites (e.g., VTTV, TCTV, and TTVV) were screened to enable Cas12a recognition. A single-stranded DNA (ssDNA) reporter labeled with 6-carboxyfluorescein (FAM) and biotin was designed for lateral flow detection. Fluorescent reporter molecules labeled with HEX–BHQ1 were used for fluorescence assays. All primer, crRNA, and reporter sequences are listed in Table 2.

### 2.3. RPA Assay Establishment

The RPA reaction was performed using the TwistAmp Basic Kit (TwistDx Limited, Maidenhead, UK) according to the manufacturer’s instructions. Each reaction was prepared in a final volume of 50 μL containing 29.5 μL rehydration buffer, 2.5 μL each of the forward and reverse primers (10 μM), 2 μL DNA template, 11 μL RNase-free water, and 2.5 μL magnesium acetate (MgOAc). MgOAc was preloaded in the tube cap and centrifuged before incubation to initiate the reaction. The mixture was incubated at 39 °C for 30 min.

The amplification products were purified using phenol:chloroform:isoamyl alcohol (25:24:1) extraction and analyzed by 1.5% agarose gel electrophoresis. Agarose gel electrophoresis was performed using a gel electrophoresis system (Bio-Rad Laboratories, Hercules, CA, USA). Bands were visualized under UV light using a gel documentation system (Universal Hood III; Bio-Rad Laboratories, Hercules, CA, USA).

RPA products were purified only before agarose gel electrophoresis analysis. For the subsequent CRISPR/Cas12a–LFA detection, crude RPA products were used directly without purification unless otherwise stated.

### 2.4. CRISPR/Cas12a Detection System Establishment

The CRISPR/Cas12a reaction (20 μL) included 2 μL 10× NEB Buffer 2.1 (New England Biolabs, Ipswich, MA, USA), 0.4 μL LbaCas12a (5 μM), 0.4 μL crRNA (10 μM), 2 μL reporter molecule (10 μM), 0.5 μL RNase inhibitor (40 U/μL), 0.5 μL dithiothreitol (DTT) (0.1 mmol/L), and 12.2 μL nuclease-free water. RPA product (2 µL) was added as a template, and ddH_2_O was used as the negative control.

After incubation under the appropriate reaction conditions, 30 μL of nuclease-free water was added to the reaction mixture for lateral flow detection. A commercial Cas12/13 nucleic acid detection lateral flow strip compatible with FAM/biotin-labeled ssDNA reporters (Beijing Baoying Tonghui Biotechnology Co., Ltd., Beijing, China) was inserted into the tube, and the result was read within 10 min according to the manufacturer’s instructions. The presence of a red band at the test line (T line) was interpreted as a positive result, regardless of the intensity or presence of the control line (C line). The presence of a red C line without a T line was interpreted as a negative result, whereas the absence of both C and T lines was considered invalid.

### 2.5. RPA Primer and crRNA Screening

Primer specificity was evaluated using genomic DNA from the 30 grape-associated fungal pathogens listed in Table 1. ddH_2_O was used as the negative control, and the positive control supplied in the TwistAmp Basic Kit was included. Amplification performance was evaluated using agarose gel electrophoresis based on the band clarity, expected size, and absence of non-specific amplification.

Candidate crRNAs were screened using LFA and fluorescence assays. Fluorescence assays were performed using HEX–BHQ1-labeled ssDNA reporters, and fluorescence signals were monitored using a qPCR instrument (Azure Cielo™ 3; Azure Biosystems, Inc., Dublin, CA, USA). The optimal crRNAs were selected based on LFA strip signal clarity, a short time to reach the fluorescence plateau, strong signal intensity, and reproducibility.

### 2.6. RPA Reaction Optimization

RPA conditions were optimized using a single-factor controlled variable method. The reaction time was fixed at 35 min, and different temperatures (33, 35, 37, 39, 41, 43, and 45 °C) were set. Under otherwise unchanged reaction conditions, amplification performance was compared at different temperatures to determine the optimal reaction temperature. At the optimal temperature, different reaction times (5, 10, 15, 20, 25, 30, 35, and 40 min) were set to screen the optimal reaction time. Amplification products under each condition were analyzed using agarose gel electrophoresis after purification, and ddH_2_O was used instead of the DNA template as the negative control. The optimal conditions were determined based on the clarity and relative brightness of the target bands, with priority given to conditions that produced clearer and brighter bands without non-specific bands.

### 2.7. CRISPR/Cas12a Reaction Optimization

A single-factor controlled variable method was used to optimize the CRISPR/Cas12a reaction system and conditions. Under a preset initial reaction time, the optimal reaction temperature and time were screened sequentially. After determining the optimal reaction temperature and time, the Cas12a protein concentration and crRNA:Cas12a ratio were optimized. Temperatures of 33, 35, 37, 39, 41, 43, and 45 °C were tested for *Ilyonectria* sp., and temperatures of 31, 33, 35, 37, 39, 41, and 43 °C were tested for *Dactylonectria* spp. Reaction times (5, 10, 15, 20, 25, 30, 35, and 40 min), crRNA:Cas12a ratios (1:4, 1:3, 1:2, 1:1, 2:1, 3:1, and 4:1), and Cas12a concentrations (25, 50, 75, 100, 125, 150, 175, and 200 nM) were also evaluated. For each condition, ddH_2_O was used instead of the DNA template as the negative control, and the optimal conditions were selected based on the LFA strip results (a clear T line signal in positive reactions, no T line in the negative control, and good reproducibility). The LFA results were interpreted according to the criteria described in Section 2.4.

### 2.8. Sensitivity Assay

DNA templates were prepared from representative strains of the two genera to determine the sensitivity of the RPA-CRISPR/Cas12a detection method. DNA samples from *Ilyonectria liriodendri* and *Dactylonectria alcacerensis* strains were selected as templates. The genomic DNA samples were 10-fold serially diluted to obtain DNA templates at different concentrations. ddH_2_O was used instead of the DNA template as the negative control. The DNA concentrations of *Dactylonectria alcacerensis* and *Ilyonectria liriodendri* were determined. The concentrations of diluted *D. alcacerensis* DNA templates were 80 ng/μL, 8 ng/μL, 800 pg/μL, 80 pg/μL, 8 pg/μL, 800 fg/μL, 80 fg/μL, 8 fg/μL, 800 ag/μL, and 80 ag/μL. The concentrations of diluted *I. liriodendri* DNA templates were 360 ng/μL, 36 ng/μL, 3.6 ng/μL, 360 pg/μL, 36 pg/μL, and 3.6 pg/μL.

The LFA results were interpreted according to the criteria described in Section 2.4. The sensitivity was defined as the lowest template concentration that produced a positive result.

### 2.9. Specificity Assay

To determine the specificity of the detection method, genomic DNA samples from 30 grape disease-related pathogenic fungi listed in Table 1, including 5 black-foot disease-related pathogens, were used as templates. Each template was tested following the established RPA-CRISPR/Cas12a workflow, and ddH_2_O was used instead of the DNA template as a negative control. The LFA results were interpreted according to the criteria described in Section 2.4. Specificity was evaluated based on positive results for target taxa and negative results for non-target taxa among the tested strains.

### 2.10. Validation Using Suspected Diseased Grapevine Samples Collected in the Field

To evaluate the applicability of the developed assay to field samples, suspected diseased grapevine samples collected in the field were tested using the optimized RPA–CRISPR/Cas12a–LFA detection system. The samples were obtained from two-year-old ‘Summer Black’ grapevines in Shenyang, Liaoning Province, China, and two-year-old ‘Shine Muscat’ grapevines in Beizhen, Liaoning Province, China. The symptomatic ‘Summer Black’ vines showed leaf yellowing, blackened roots, and black necrosis of the vascular tissues in transverse sections, whereas the symptomatic ‘Shine Muscat’ vines showed vine decline, poor growth, leaf curling, and varying degrees of necrosis in transverse sections of roots and stems. For each cultivar, two original symptomatic samples, including one root sample and one stem sample, were collected. From these original samples, a total of 10 tissue subsamples from ‘Summer Black’ and 13 tissue subsamples from ‘Shine Muscat’ were selected from different symptomatic or suspected diseased tissues and used for DNA extraction and subsequent detection.

The original samples had been diagnosed as being associated with grapevine black-foot disease and other grapevine trunk diseases based on fungal isolation, culture characteristics, and morphological identification. DNA was extracted from each of the 23 tissue subsamples using the CTAB method. Each DNA sample was tested in three technical replicates using the optimized detection system. Genomic DNA samples of *I. liriodendri* and *D. alcacerensis* were used as positive controls, and ddH_2_O was used as the negative control. The LFA results were interpreted according to the criteria described in Section 2.4.

## 3. Results

### 3.1. RPA Primer and crRNA Validation

**Figure 1:**
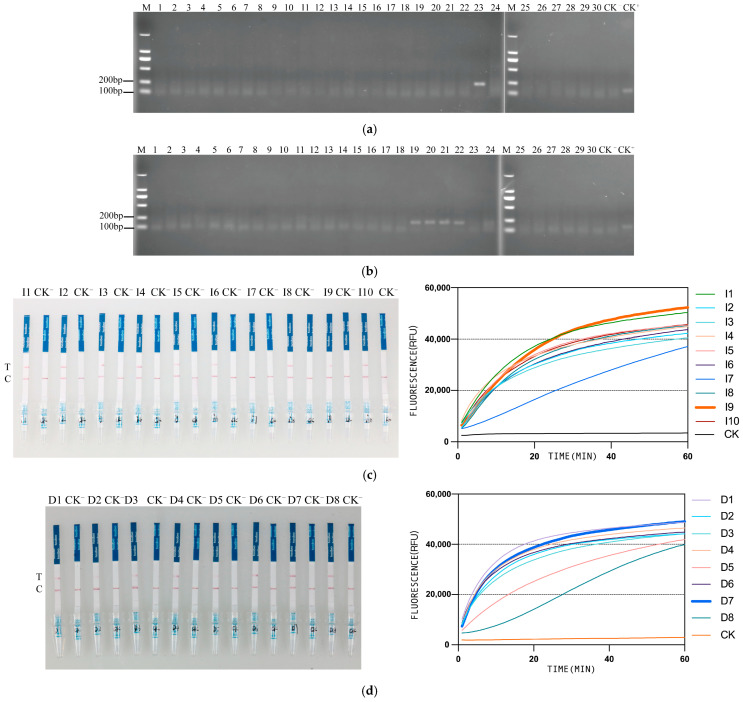
Design and screening of RPA primers and crRNA. (**a**): Screening of primers for *Ilyonectria* sp.; (**b**): screening of primers for *Dactylonectria* spp.; (**c**): crRNA screening for *Ilyonectria* sp. using the strip method and crRNA screening for *Ilyonectria* sp. using the fluorescence method; (**d**): crRNA screening for *Dactylonectria* spp. using the strip method and crRNA screening for *Dactylonectria* spp. using the fluorescence method. M: DL2000 DNA Marker; 1: *Cadophora luteo*-*olivacea*; 2: *Cad. malorum*; 3: *Cad. sabaouae*; 4: *Phaeoacremonium iranianum*; 5: *Paraeutypella citricola*; 6: *Neocosmospora solani*; 7: *Neo. falciformis*; 8: *Fusarium annulatum*; 9: *F. oxysporum*; 10: *F. proliferatum*; 11: *F. acuminatum*; 12: *Diaporthe eres*; 13: *Dia. sojae*; 14: *Rosellinia necatrix*; 15: *Coniella vitis*; 16: *Cylindrocladiella lageniformis*; 17: *Cyl. peruviana*; 18: *Cyl. viticola*; 19: *Dactylonectria alcacerensis*; 20: *Dac. macrodidyma*; 21: *Dac. novozelandica*; 22: *Dac. torresensis*; 23: *Ilyonectria liriodendri;* 24: *Botryosphaeria dothidea*; 25: *Lasiodiplodia theobromae*; 26: *Neopestalotiopsis* sp.; 27: *Cladosporium* sp.; 28: *Colletotrichum viniferum*; 29: *Col. acutatum*; 30: *Col. falcatum*; CK^−^: negative control; CK^+^: positive control. D1–D8 indicate the candidate crRNAs designed for *Dactylonectria*, corresponding to D-crRNA-1 to D-crRNA-8, respectively; I1–I10 indicate the candidate crRNAs designed for *Ilyonectria*, corresponding to I-crRNA-1 to I-crRNA-10, respectively. Non-adjacent lanes are shown; all samples were amplified under identical conditions. Each assay was tested in three technical replicates.

Candidate RPA primers targeting the *HIS* and *TUB* genes were screened and validated for *Ilyonectria* sp. and *Dactylonectria* spp., respectively. For *Ilyonectria* sp., the primer pair HIS-il-95-124F/HIS-il-318R produced clearer and brighter target bands with better specificity. For *Dactylonectria* spp., TUB-dac-101-130F/TUB-dac-260R showed better amplification performance and specificity. Thus, these primer pairs were selected for subsequent experiments (Figure 1a,b).

After identifying the optimal primers, the recognition performance of candidate crRNAs was validated in the CRISPR/Cas12a system. I-crRNA-9 of *Ilyonectria* sp. and D-crRNA-7 of *Dactylonectria* spp. produced stronger and more stable fluorescence signal outputs and consistently generated positive bands on the test strips; therefore, they were selected as the optimal crRNAs for subsequent experiments (Figure 1c,d).

### 3.2. RPA-CRISPR/Cas12a System Optimization

**Figure 2:**
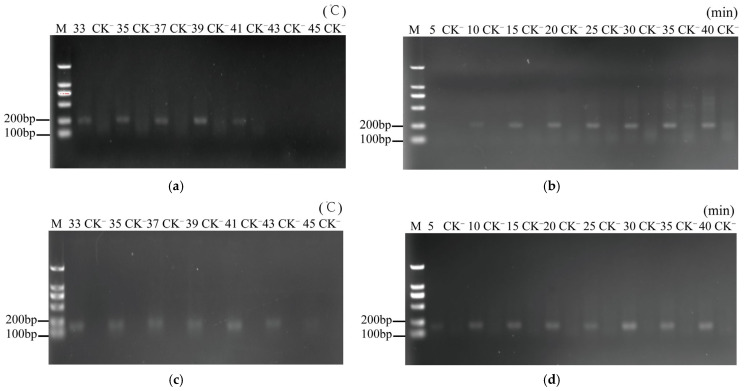
Optimization of RPA reaction conditions. (**a**): Temperature optimization of the RPA system for *Ilyonectria* sp.; (**b**): Time optimization of the RPA system for *Ilyonectria* sp.; (**c**): Temperature optimization of the RPA system for *Dactylonectria* spp.; (**d**): Time optimization of the RPA system for *Dactylonectria* spp.; CK^−^ is the negative control with sterile water.

RPA reaction conditions were optimized using a single-factor controlled variable method, and the amplification products were analyzed using agarose gel electrophoresis. When screening the RPA reaction temperature, the amplification bands of *Ilyonectria* sp. and *Dactylonectria* spp. gradually became clearer with increasing temperature and then weakened until disappearing. When screening the RPA reaction time, the amplification bands of both targets gradually increased as the reaction time was prolonged and stabilized after a certain time. Optimal reaction conditions were determined in combination with the band changes. As shown in Figure 2, the optimal RPA reaction temperature and amplification time for *Ilyonectria* sp. were 39 °C and 30 min, respectively, and those for *Dactylonectria* spp. were 41 °C and 30 min, respectively.

**Figure 3:**
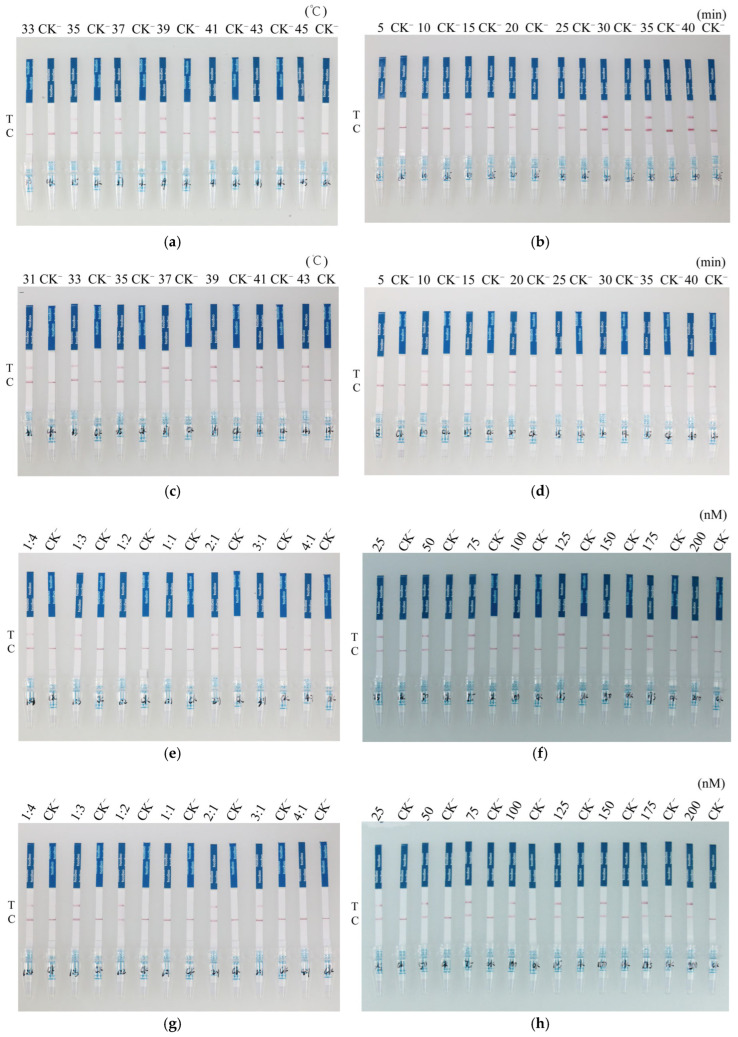
Optimization of the CRISPR reaction system. (**a**): Temperature optimization of the CRISPR system for *Ilyonectria* sp.; (**b**): Time optimization of the CRISPR system for *Ilyonectria* sp.; (**c**): Temperature optimization of the CRISPR system for *Dactylonectria* spp.; (**d**): Time optimization of the CRISPR system for *Dactylonectria* spp.; (**e**): Optimization of the crRNA:Cas12a ratio in the CRISPR system for *Ilyonectria* sp.; (**f**): Optimization of the Cas12a concentration in the CRISPR system for *Ilyonectria* sp.; (**g**): Optimization of the crRNA:Cas12a ratio in the CRISPR system for *Dactylonectria* spp.; (**h**): Optimization of the Cas12a concentration in the CRISPR system for *Dactylonectria* spp.; CK^−^ is the negative control with sterile water. Each sample was tested in three technical replicates.

The CRISPR/Cas12a reaction conditions were optimized based on the strip detection results. When screening the reaction temperature, the bands on the T line bands in both assays became gradually clearer with increasing temperature and stabilized within a certain temperature range. When screening the reaction time, the detection bands of both assays gradually increased as the reaction time was prolonged and then plateaued. Based on band changes, the condition producing the most intense T-line signal was selected as the optimal reaction parameter within a reasonable range of reaction conditions. As shown in Figure 3a–d, the optimal CRISPR/Cas12a reaction temperature and time for detecting *Ilyonectria* sp. were 39 °C and 15 min, respectively, and those for detecting *Dactylonectria* spp. were 35 °C and 30 min, respectively.

The crRNA: Cas12a ratio and final Cas12a concentration were screened in combination with the experimental results, and the optimal crRNA: Cas12a ratio for both assays was 2:1. Since differences among the tested Cas12a concentrations were not substantial, the initial concentration of 100 nM was retained, as shown in Figure 3e–h.

### 3.3. Sensitivity of the RPA-CRISPR/Cas12a Detection System

**Figure 4:**
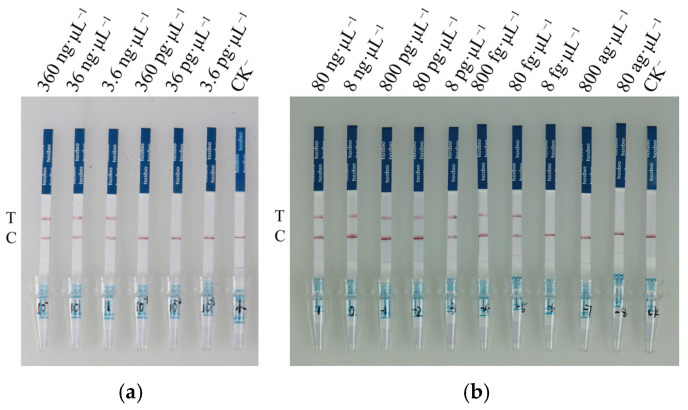
Limit of detection (LOD). (**a**) Limit of detection of the *Ilyonectria*-specific assay using *I*. *liriodendri* genomic DNA; (**b**) limit of detection of the *Dactylonectria*-specific assay using *D. alcacerensis* genomic DNA; CK^−^ is the negative control with sterile water. Each sample was tested in three technical replicates.

RPA-CRISPR/Cas12a detection was performed using genomic DNA from *I. liriodendri* and *D. alcacerensis* as templates, and the results were visualized using LFA strips. As shown in Figure 4, the lowest detectable concentration for *Ilyonectria*-targeted assay was 3.6 ng/μL, and the lowest detectable concentration for *Dactylonectria*-targeted assay was 80 fg/μL.

### 3.4. Sensitivity of the RPA-CRISP/Cas12a Detection System

**Figure 5:**
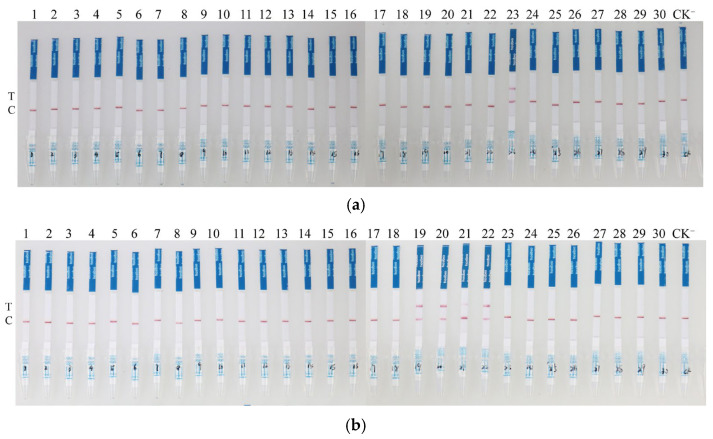
Specificity analysis. (**a**): Specific detection of *Ilyonectria* sp.; (**b**): Specific detection of *Dactylonectria* spp. 1: *Cadophora luteo-olivacea*; 2: *Cad. malorum*; 3: *Cad. sabaouae*; 4: *Phaeoacremonium iranianum*; 5: *Paraeutypella citricola*; 6: *Neocosmospora solani*; 7: *Neo. falciformis*; 8: *Fusarium annulatum*; 9: *F. oxysporum*; 10: *F. proliferatum*; 11: *F. acuminatum*; 12: *Diaporthe eres*; 13: *Dia. sojae*; 14: *Rosellinia necatrix*; 15: *Coniella vitis*; 16: *Cylindrocladiella lageniformis*; 17: *Cyl. peruviana*; 18: *Cyl. viticola*; 19: *Dactylonectria alcacerensis*; 20: *Dac. macrodidyma*; 21: *Dac. novozelandica*; 22: *Dac. torresensis*; 23: *Ilyonectria liriodendri*; 24: *Botryosphaeria dothidea*; 25: *Lasiodiplodia theobromae*; 26: *Neopestalotiopsis* spp.; 27: *Cladosporium*; 28: *Colletotrichum viniferum*; 29: *Col. acutatum*; 30: *Col. falcatum*; CK^−^: negative control. Each sample was tested in three technical replicates.

Using the optimal primers and crRNAs for grapevine black-foot disease pathogens, RPA-CRISPR/Cas12a detection was performed using genomic DNA from 30 grape disease-related fungi listed in Table 1, including pathogens associated with grapevine black-foot disease, and the detection results were presented using LFA strips. As shown in Figure 5, the assay showed a positive reaction only for the DNA of the target pathogens.

### 3.5. Validation Using Symptomatic Grapevine Samples

To further evaluate the applicability of the established assays to field-collected symptomatic samples, two batches of field-collected symptomatic grapevine samples were tested using the optimized *Ilyonectria*-specific and *Dactylonectria*-specific RPA-CRISPR/Cas12a-LFA detection systems.

**Figure 6:**
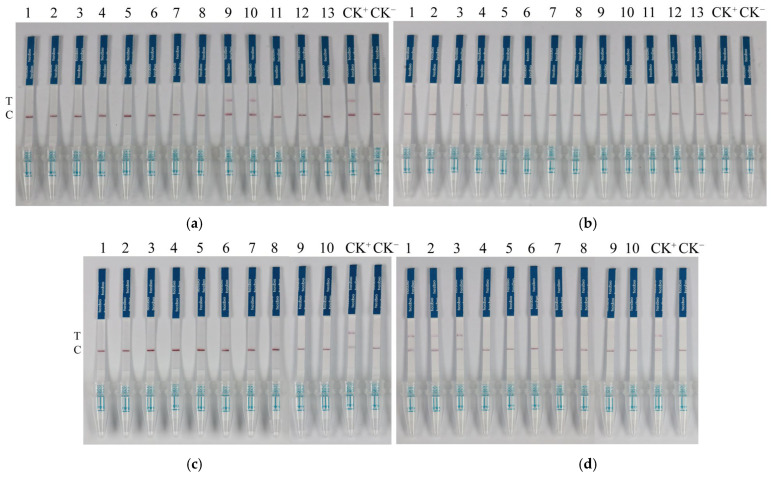
Detection of symptomatic grapevine samples using the RPA-CRISPR/Cas12a-LFA. (**a**) Detection of ‘Shine Muscat’ samples using the *Ilyonectria*-specific RPA–CRISPR/Cas12a–LFA; (**b**) Detection of ‘Shine Muscat’ samples using the *Dactylonectria*-specific RPA–CRISPR/Cas12a–LFA; (**c**)Detection of ‘Summer Black’ samples using the *Ilyonectria*-specific RPA–CRISPR/Cas12a–LFA assay; (**d**) Detection of ‘Summer Black’ samples using the *Dactylonectria*-specific RPA–CRISPR/Cas12a–LFA. In panels (**a**,**b**), 1–13 indicate the tested tissue subsamples; in panels (**c**,**d**), 1–10 indicate the tested tissue subsamples.; CK^+^: positive control using genomic DNA of *Ilyonectria liriodendri* or *Dactylonectria alcacerensis*, depending on the corresponding assay; CK^−^: negative control using ddH_2_O. The experiment was performed with three technical replicates, and representative results are shown.

The first batch consisted of thirteen tissue subsamples obtained from symptomatic ‘Shine Muscat’ grapevines. Each sample was tested using both the *Ilyonectria*-specific and *Dactylonectria*-specific assays. Genomic DNA of *I. liriodendri* and *D. alcacerensis* was used as the positive control for the corresponding detection systems, and ddH_2_O was used as the negative control. As shown in Figure 6a, two of the thirteen samples produced positive LFA signals in the *Ilyonectria*-specific assay. No positive signal was observed in the *Dactylonectria*-specific assay for this batch of samples (Figure 6b). These results indicated that *Ilyonectria*-associated DNA was detected in the ‘Shine Muscat’ samples under the tested conditions.

The second batch consisted of ten tissue subsamples obtained from symptomatic ‘Summer Black’ grapevines. These samples were also tested using both detection systems. As shown in Figure 6c, no positive signal was observed in the *Ilyonectria*-specific assay. In the *Dactylonectria*-specific assay, three of the ten samples produced positive LFA signals (Figure 6d). These results indicated that *Dactylonectria*-associated DNA was detected in the ‘Summer Black’ samples under the tested conditions.

## 4. Discussion

This study established two RPA–CRISPR/Cas12a–LFAs for the rapid and visual detection of selected species associated with grapevine black-foot disease within *Ilyonectria* and *Dactylonectria* previously reported in China. The assays were not designed as comprehensive genus-level detection systems covering every species within these genera. Compared with conventional PCR and qPCR-based methods, the assays do not require thermal cycling equipment during amplification and detection and provide a visual readout within a short turnaround time. The current workflow reduces the requirement for thermocyclers and fluorescence detection equipment during amplification and readout, facilitating visual result interpretation after DNA extraction. However, the current DNA extraction step relies on CTAB-based methods requiring centrifugation and organic solvents, which limits deployment in fully resource-limited settings. The development of a rapid DNA extraction method compatible with on-site operation would be required before the workflow could be considered suitable for field application.

For the *Ilyonectria*-targeted assay, the BLAST analysis (National Center for Biotechnology Information, Bethesda, MD, USA; https://blast.ncbi.nlm.nih.gov/Blast.cgi, accessed on 4 June 2026) retrieved sequences annotated as *I. liriodendri* and *D. macrodidyma*. Further sequence comparison showed that the retrieved sequence annotated as *D. macrodidyma* was identical to the ex-type sequence of *I. liriodendri* but differed from the ex-type sequence of *D. macrodidyma*, suggesting that the corresponding database record may have been misannotated. Therefore, this record cannot be regarded as reliable evidence of cross-reactivity between the assay and an authenticated *D. macrodidyma* isolate. The available database analysis supports the predicted specificity of the *Ilyonectria*-targeted assay for *I. liriodendri*, although further validation using additional authenticated non-target isolates is still required.

Primer-BLAST analysis of the *Dactylonectria*-targeted assay identified 13 potential non-target hits in addition to the four target species defined in this study (*Dactylonectria alcacerensis*, *D. torresensis*, *D. macrodidyma*, and *D. novozelandica*) (Table S1). On this basis, the potential risk of cross-recognition was further analyzed by combining the crRNA spacer BLAST results with PAM-site compatibility. These hits included taxa that have not yet been reported in studies related to grapevine black-foot disease in China, as well as non-target fungi that are taxonomically closely related to the target fungi.

Among the non-target fungi that are taxonomically closely related to the target fungi, a literature review showed that most taxa have only been reported as isolates from non-grapevine hosts or other environmental samples. Among them, only *Dactylonectria* ecuadoriensis was retrieved from a grapevine root-associated microbiome sequencing record. No direct evidence was found showing that these non-target taxa have been isolated or clearly detected in internal grapevine tissues or grapevine-associated environments in China. These hits mainly reflect sequence similarity at the database level and do not directly indicate that they would interfere with the detection of the target grapevine black-foot pathogens-associated DNA in this study.

This assay can generate a signal only when RPA by both primers, crRNA spacer matching, and PAM-site compatibility are simultaneously satisfied. For non-target fungi that show similarity only in the primer-binding regions, CRISPR recognition can provide an additional screening step. However, further analysis in this study showed that some potential non-target taxa can simultaneously satisfy the above conditions. Therefore, if the corresponding DNA is present in the sample to be tested, the possibility of generating a positive signal cannot be completely excluded. Considering the existing database records, sample sources, and pretreatment methods, the actual risk of interference from these non-target taxa may be relatively limited under the detection workflow used in this study for internal diseased tissues from symptomatic grapevines. When testing soil, roots or asymptomatic tissues, the presence of environmental or saprophytic species in the genera *Ilyonectria* and *Dactylonectria* may lead to false positive diagnoses. Further validation using additional field samples and non-target isolates is still required to assess the practical specificity of the *Dactylonectria*-targeted assay.

Therefore, for samples such as soil, nursery substrates, root-surface materials, or asymptomatic tissues, the results should be interpreted comprehensively in combination with the sample type, symptom expression, pathogen isolation, sequencing, or other supplementary evidence.

PCR-based methods have previously been used for the detection of grapevine black-foot disease-associated fungi. Nested PCR can detect low amounts of target DNA, and multiplex PCR enables species-specific detection [20,35]. qPCR has also been applied to the detection of *Ilyonectria* and *Dactylonectria*, offering quantitative capability but requiring a real-time PCR instrument [19]. ddPCR can provide high analytical sensitivity but relies on specialized equipment. In contrast, the RPA–CRISPR/Cas12a–LFAs developed in this study employ isothermal amplification, provide a visual readout, and complete amplification and detection steps within 1 h. The analytical sensitivity differed between the two assays developed in this study. The *Dactylonectria*-targeted assay achieved a limit of detection of 80 fg/μL, which is within the range reported for several PCR-based detection methods and indicates a high level of analytical sensitivity. By comparison, the *Ilyonectria*-targeted assay showed a higher detection limit. Nevertheless, direct comparisons of detection limits among different studies should be interpreted cautiously because the target genes, DNA templates, reaction conditions, and evaluation criteria may differ. Overall, the principal advantages of the present assays are their relatively short workflow, visual readout, and reduced reliance on specialized instrumentation during amplification and detection.

The analytical sensitivity of an RPA–CRISPR/Cas12a–LFA may be influenced by multiple factors, including primer amplification efficiency, the characteristics of the amplified region, crRNA recognition efficiency, and the use of suboptimal PAM sites within the available RPA amplicon. These factors may contribute to the difference in sensitivity between the two assays developed in this study. The limit of detection of the *Ilyonectria*-targeted assay was 3.6 ng/μL. Its applicability to samples with low pathogen biomass may therefore be limited. Further optimization, including the screening of additional primer pairs and crRNAs, the selection of more favorable PAM or near-PAM sites, and the refinement of reaction conditions, may improve analytical sensitivity.

In this study, field-collected symptomatic grapevine samples were tested using the established RPA–CRISPR/Cas12a–LFAs. The detection results were consistent with those obtained by fungal isolation, culture characteristics, and morphological identification, supporting the applicability of the assays for detecting *Ilyonectria* and *Dactylonectria* in symptomatic grapevine tissue-derived DNA samples. However, only symptomatic grapevine tissues were evaluated in the present study, with 13 and 10 tissue subsamples for the *Ilyonectria*- and *Dactylonectria*-targeted assays, respectively. These sample sizes are limited and represent preliminary field validation. Larger-scale surveys across diverse grape-growing regions and cultivars are needed to fully establish assay performance under field conditions. Additional studies are required to evaluate assay performance in asymptomatic tissues and latent infections.

Despite the promising performance of the two-step RPA-CRISPR/Cas12a-LFA system, methodological limitations remain. A primary concern in conventional two-step workflows is the need to open reaction tubes and transfer amplification products, which increases the risk of aerosol contamination. A previous study highlighted amplicon contamination during tube opening and transfer as a major obstacle to the practical application of two-step RPA-CRISPR systems in point-of-care testing [36].

To mitigate these contamination risks, recent studies have proposed one-pot (single-tube) RPA-CRISPR/Cas12a strategies that enable isothermal amplification and CRISPR detection to proceed sequentially or simultaneously in a sealed reaction system. Such designs eliminate the intermediate transfer steps and prevent amplicon exposure. For instance, a photo-controllable blocking strategy has been developed to temporarily inhibit Cas12a activity during RPA, with light activation used to initiate CRISPR detection, which reduces the contamination risk and maintains high sensitivity within 40 min [37]. Alternative approaches include the physical separation of RPA and CRISPR components in different compartments of the same tube (e.g., using oil-phase or wax barriers), enabling closed-system integration upon reaction initiation [38]. These designs have significantly improved operational safety and reproducibility, but one-pot systems face technical challenges, particularly competition between RPA and Cas12a activity in a single reaction environment, which may affect reaction kinetics and detection sensitivity [39].

Future optimization should focus on developing a detection platform that balances sensitivity, specificity, and fully closed operation. The integration of photo-regulated elements, reagent isolation strategies, and engineered crRNA components may enable the precise temporal regulation of amplification and cleavage reactions. Combining such one-pot strategies with the selected primer/crRNA sets established in this study may improve operational convenience, result stability, and contamination control. The incorporation of a rapid DNA extraction method compatible with on-site operation would also be necessary before the workflow could be considered suitable for field application.

## 5. Conclusions

In this study, we developed two RPA-CRISPR/Cas12a-LFAs for rapid visual detection of selected grapevine black-foot disease-associated species in China. The established assays completed the amplification and detection workflow within 1 h at 39–41 °C. The limits of detection were 3.6 ng/μL for the *Ilyonectria liriodendri*-targeted assay and 80 fg/μL for the *Dactylonectria*-targeted assay. The assays exhibited good specificity for the selected grapevine black-foot disease-associated species evaluated in this study, although further validation with additional authenticated non-target isolates is required. The assays showed different levels of sensitivity between the two assays, with higher analytical sensitivity for the *Dactylonectria*-targeted assay. These assays provide rapid visual detection after DNA extraction, reducing the requirement for specialized instrumentation during amplification and readout, but are not currently suitable for fully field-deployable application without further optimization of DNA extraction methods.

## Figures and Tables

**Table 1:** Fungal taxa and associated diseases of grape-associated fungi used in this study.

No.	Fungal Taxon	Associated Disease	References
1	*Cadophora luteo-olivacea*	Grapevine esca disease complex	[30]
2	*Cadophora malorum*	Grapevine esca disease complex	[30]
3	*Cadophora sabaouae*	Grapevine esca disease complex	[31]
4	*Phaeoacremonium iranianum*	Grapevine esca disease complex	[30]
5	*Paraeutypella citricola*	Eutypa dieback	[31]
6	*Neocosmospora solani*	Grapevine Fusarium root rot	[30]
7	*Neocosmospora falciformis*	Grapevine Fusarium root rot	[30]
8	*Fusarium annulatum*	Grapevine Fusarium root rot	[31]
9	*Fusarium oxysporum*	Grapevine Fusarium root rot	[30]
10	*Fusarium proliferatum*	Grapevine Fusarium root rot	[30]
11	*Fusarium acuminatum*	Grapevine Fusarium root rot	[30]
12	*Diaporthe eres*	Phomopsis dieback	[30]
13	*Diaporthe sojae*	Phomopsis dieback	[32]
14	*Rosellinia necatrix*	Grapevine white root rot	[30]
15	*Coniella vitis*	Grapevine white rot	[30]
16	*Cylindrocladiella lageniformis*	Grapevine black-foot disease	[2]
17	*Cylindrocladiella peruviana*	Grapevine black-foot disease	[12]
18	*Cylindrocladiella viticola*	Grapevine black-foot disease	[32]
19	*Dactylonectria alcacerensis*	Grapevine black-foot disease	[2]
20	*Dactylonectria macrodidyma*	Grapevine black-foot disease	[2]
21	*Dactylonectria novozelandica*	Grapevine black-foot disease	[15]
22	*Dactylonectria torresensis*	Grapevine black-foot disease	[2]
23	*Ilyonectria liriodendri*	Grapevine black-foot disease	[32]
24	*Botryosphaeria dothidea*	Botryosphaeria dieback	[33]
25	*Lasiodiplodia theobromae*	Botryosphaeria dieback	[33]
26	*Neopestalotiopsis* sp.	Grapevine shoot blight	-
27	*Cladosporium* sp.	Grapevine shoot blight	[32]
28	*Colletotrichum viniferum*	Grapevine anthracnose	[32]
29	*Colletotrichum acutatum*	Grapevine anthracnose	[32]
30	*Colletotrichum falcatum*	Grapevine anthracnose	-

**Table 2:** RPA primers, crRNAs, and reporter sequences used for the detection of *Ilyonectria* and *Dactylonectria* in this study.

Name	Sequence (5′-3′)
HIS-il-95-124F	ACGCGTCTTGCAACATCTATCTTCATCACA
HIS-il-318R	GACCTGTGCCGGGTGTGTTAGAGTGAATGC
TUB-dac-101-130F	CCGCTGCAGCATTTCCACCGCCTCGAGCAA
TUB-dac-260R	TACCCTATCCACGCGTTGTTAGAATCTCCG
I-crRNA-9	UAAUUUCUACUAAGUGUAGAU UCUACCGGUGGUGUCAAGAAGCCU
D-crRNA-7	UAAUUUCUACUAAGUGUAGAU GUGCAAUAUAGGUCCACCUCCAGA
CR-DNA-FB-2	FAM-TTTATTT-Biotin
CR-DNA-HQ	HEX-TTTTTTTATTTTTTT-BHQ1

## Data Availability

The data presented in this study are available in the article and Supplementary Materials.

## Supplementary Materials

The following supporting information can be downloaded at: https://www.mdpi.com/article/10.3390/jof12070455/s1. Table S1: In silico evaluation of potential non-target hits for the *Dactylonectria*-specific RPA–CRISPR/Cas12a assay based on RPA primer-pair matching, crRNA spacer similarity, and PAM compatibility [40].
