# Supplementary material for: A Rapid Recombinase Polymerase Amplification–CRISPR/Cas12a Assay for Detecting Grapevine Black-Foot Pathogens
Source: J Fungi (Basel). 2026 Jun 23;12(7):455. doi: 10.3390/jof12070455 (PMC13413064; doi:10.3390/jof12070455)
Supplement: Supplementary file 1 [file jof-12-00455-s001.zip › jof-4243364-supplementary.pdf]

Table S1 In silico evaluation of potential non-target hits for the *Dactylonectria*-specific RPA–CRISPR/Cas12a assay based on RPA primer-pair matching, crRNA spacer similarity, and PAM compatibility.

No.	Taxon	Forward primer match	Reverse primer match	Accession No.	crRNA spacer match and Mismatch positions	PAM type	Reported association with grapevine	Reference
1	*Thelonectria lucida*	30/30	30/30	KJ022320.1	24/24	Non-canonical or potentially suboptimal PAM (TCTT)	No published reports were identified of its isolation from grapevine tissues or detection in grapevine-associated samples.	-
				AY677259.1	21/24 nt, P1, P3 and P4 at the PAM-proximal end	Canonical PAM (TTTC)		
				KC153819.1	21/24 nt, P1, P3 and P4 at the PAM-proximal end	Canonical PAM (TTTC)		
				KJ022333.1	23/24 nt, P1 at the PAM-proximal end	Canonical PAM (TTTC)		
				AY677259.1	20/24 nt, P1,P2,P3 and P4 at the PAM-proximal end	Non-canonical or potentially suboptimal PAM (TTCC)		
				KF569865.1	21/24 nt, P1, P3 and P4 at the PAM-proximal end	Canonical PAM (TTTC)		
				KF569866.1	21/24 nt, P1, P3 and P4 at the PAM-proximal end	Canonical PAM (TTTC)		

				KJ022316.1	20/24 nt, P1,P2,P3 and P4 at the PAM-proximal end	Non-canonical or potentially suboptimal PAM (TTCC)		
				KJ022319.1	21/24 nt, P1, P3 and P4 at the PAM-proximal end	Canonical PAM (TTTC)		
				KJ022318.1	21/24 nt, P1, P3 and P4 at the PAM-proximal end	Canonical PAM (TTTC)		
				KJ022317.1	21/24 nt, P1, P3 and P4 at the PAM-proximal end	Canonical PAM (TTTC)		
				KJ022314.1	20/24 nt, P1,P2,P3 and P4 at the PAM-proximal end	Canonical PAM (TTTC)		
				KC153781.1	21/24 nt, P1, P3 and P4 at the PAM-proximal end	Canonical PAM (TTTC)		
				KC153822.1	21/24 nt, P1, P3 and P4 at the PAM-proximal end	Canonical PAM (TTTC)		
				KJ022315.1	21/24 nt, P1, P3 and P4 at the PAM-proximal end	Canonical PAM (TTTC)		

				KC153811.1	21/24 nt, P1, P3 and P4 at the PAM-proximal end	Canonical PAM (TTTC)		
				KJ022321.1	20/24 nt, P1,P2,P3 and P4 at the PAM-proximal end	Canonical PAM (TTTC)		
2	*Thelonectria westlandica*	30/30	30/30	KF569871.1	24/24	Non-canonical or potentially suboptimal PAM (TCTT)	No published reports were identified of its isolation from grapevine tissues or detection in grapevine-associated samples.	-
				HM352868.1	20/24 nt, P1,P2,P3 and P4 at the PAM-proximal end	Canonical PAM (TTTC)		
				KF569870.1	20/24 nt, P1,P2,P3 and P4 at the PAM-proximal end	Canonical PAM (TTTC)		
				HM484610.1	20/24 nt, P1,P2,P3 and P4 at the PAM-proximal end	Canonical PAM (TTTC)		
				KF569872.1	20/24 nt, P1,P2,P3 and P4 at the PAM-proximal end	Canonical PAM (TTTC)		
3	*Thelonectria theobromicola*	30/30	28/30	KJ022333.1	23/24 nt, P1 at the PAM-proximal end	Non-canonical or potentially suboptimal PAM (TCTT)	No published reports were identified of its isolation from grapevine tissues or	-

				KJ022334.1	21/24 nt, P1, P3 and P4 at the PAM-proximal end	Non-canonical or potentially suboptimal PAM (TTTT)	detection in grapevine-associated samples.	
				EF607062.1	21/24 nt, P1, P3 and P4 at the PAM-proximal end	Non-canonical or potentially suboptimal PAM (TTTT)		
4	*Dactylonectria anthuriicola*	30/30	28/30	JF735430.1	23/24 nt, P1 at the PAM-proximal end	Non-canonical or potentially suboptimal PAM (TCTT)	No published reports were identified of its isolation from grapevine tissues or detection in grapevine-associated samples.	-
5	*Dactylonectria dicranopteridis*	30/30	28/30	PV221991.1	23/24 nt, P1 at the PAM-proximal end	Non-canonical or potentially suboptimal PAM (TCTT)	No pub-lished re-ports were identified of its isolation from grape-vine tissues or detection in grape-vine-associated samples.	-
6	*Dactylonectria valentina*	30/30	28/30	KY676875.1	23/24 nt, P1 at the PAM-proximal end	Non-canonical or potentially suboptimal PAM (TCTT)	No pub-lished re-ports were identified of its isolation from grape-vine tissues or detection in grape-vine-associated samples.	-
				MK409881.1	23/24 nt, P1 at the PAM-proximal end	Non-canonical or potentially suboptimal PAM (TCTT)		
7	*Dactylonectria ecuadoriensis*	30/30	28/30	MK409883.1	23/24 nt, P1 at the PAM-proximal end	Non-canonical or potentially suboptimal PAM (TCTT)	Detected in grapevine root-associated fungal communities in France by ITS2	[40]

				MF683642.1	23/24 nt, P1 at the PAM-proximal end	Non-canonical or potentially suboptimal PAM (TCTT)	metabarcoding; no published reports of its isolation from grapevine tissues were identified.	
				MF683638.1	23/24 nt, P1 at the PAM-proximal end	Non-canonical or potentially suboptimal PAM (TCTT)		
				MF683640.1	23/24 nt, P1 at the PAM-proximal end	Non-canonical or potentially suboptimal PAM (TCTT)		
				MF683639.1	23/24 nt, P1 at the PAM-proximal end	Non-canonical or potentially suboptimal PAM (TCTT)		
				MF683641.1	23/24 nt, P1 at the PAM-proximal end	Non-canonical or potentially suboptimal PAM (TCTT)		
				MF683637.1	23/24 nt, P1 at the PAM-proximal end	Non-canonical or potentially suboptimal PAM (TCTT)		
8	*Dactylonectria hispanica*	29/30	28/30	KY676876.1	23/24 nt, P1 at the PAM-proximal end	Non-canonical or potentially suboptimal PAM (TCTT)	No published reports were identified of its isolation from grapevine tissues or detection in grapevine-associated samples.	-

9	*Dactylonectria amazonica*	30/30	28/30	MF683644.1	22/24 nt, P1 and P5 at the PAM-proximal end	Non-canonical or potentially suboptimal PAM (TCTT)	No pub-lished re-ports were identified of its isolation from grape-vine tissues or detection in grape-vine-associated samples.	-
				MF683643.1	22/24 nt, P1 and P5 at the PAM-proximal end	Non-canonical or potentially suboptimal PAM (TCTT)		
10	*Dactylonectria lehmanniae*	30/30	28/30	PQ213372.1	22/24 nt, P1 and P7 at the PAM-proximal end	Non-canonical or potentially suboptimal PAM (TCTT)	No pub-lished re-ports were identified of its isolation from grape-vine tissues or detection in grape-vine-associated samples.	-
11	*Ilyonectria destructans*	30/30	30/30	-	-	-	Reported as grapevine black-foot pathogens outside China, but not yet reported in China.	[1]
12	*Dactylonectria vitis*	30/30	28/30	-	-	-	Reported as grapevine black-foot pathogens outside China, but not yet reported in China.	[1]
13	*Dactylonectria pauciseptata*	30/30	28/30	-	-	-	Reported as grapevine black-foot pathogens outside China, but not yet reported in China.	[1]

The numbers of mismatches between each potential non-target sequence and the forward primer, reverse primer, and crRNA spacer are shown separately. PAM sequences adjacent to the corresponding protospacer regions were examined to assess their potential compatibility with Cas12a recognition.

These database-level matches represent a theoretical risk of false-positive detection. Our current experimental validation does not include these specific non-target taxa, and therefore the possibility of cross-reactivity cannot be completely excluded. The presence of matching primer-binding regions and a potentially compatible PAM indicates only a theoretical possibility of cross-reactivity and does not necessarily result in detectable amplification or Cas12a-mediated signal generation under the experimental conditions.
